# Single-cell characterization of a model of poly I:C-stimulated peripheral blood mononuclear cells in severe asthma

**DOI:** 10.1186/s12931-021-01709-9

**Published:** 2021-04-26

**Authors:** Ailu Chen, Maria P. Diaz-Soto, Miguel F. Sanmamed, Taylor Adams, Jonas C. Schupp, Amolika Gupta, Clemente Britto, Maor Sauler, Xiting Yan, Qing Liu, Gustavo Nino, Charles S. Dela Cruz, Geoffrey L. Chupp, Jose L. Gomez

**Affiliations:** 1 Section of Pulmonary, Critical Care and Sleep Medicine, Department of Internal Medicine, Yale University School of Medicine, 300 Cedar Street (S419 TAC), New Haven, CT 06520-8057 USA; 2 Department of Oncology, Clínica Universidad de Navarra, Pamplona, Spain; 3 Program of Immunology and Immunotherapy, Center for Applied Medical Research, University of Navarra, Pamplona, Spain; 4 Division of Pediatric Pulmonary and Sleep Medicine, Children’s National Hospital, Department of Pediatrics, George Washington University, Washington, DC USA

## Abstract

**Background:**

Asthma has been associated with impaired interferon response. Multiple cell types have been implicated in such response impairment and may be responsible for asthma immunopathology. However, existing models to study the immune response in asthma are limited by bulk profiling of cells. Our objective was to Characterize a model of peripheral blood mononuclear cells (PBMCs) of patients with severe asthma (SA) and its response to the TLR3 agonist Poly I:C using two single-cell methods.

**Methods:**

Two complementary single-cell methods, DropSeq for single-cell RNA sequencing (scRNA-Seq) and mass cytometry (CyTOF), were used to profile PBMCs of SA patients and healthy controls (HC). Poly I:C-stimulated and unstimulated cells were analyzed in this study.

**Results:**

PBMCs (n = 9414) from five SA (n = 6099) and three HC (n = 3315) were profiled using scRNA-Seq. Six main cell subsets, namely CD4 + T cells, CD8 + T cells, natural killer (NK) cells, B cells, dendritic cells (DCs), and monocytes, were identified. CD4 + T cells were the main cell type in SA and demonstrated a pro-inflammatory profile characterized by increased JAK1 expression. Following Poly I:C stimulation, PBMCs from SA had a robust induction of interferon pathways compared with HC. CyTOF profiling of Poly I:C stimulated and unstimulated PBMCs (n = 160,000) from the same individuals (SA = 5; HC = 3) demonstrated higher CD8 + and CD8 + effector T cells in SA at baseline, followed by a decrease of CD8 + effector T cells after poly I:C stimulation.

**Conclusions:**

Single-cell profiling of an in vitro model using PBMCs in patients with SA identified activation of pro-inflammatory pathways at baseline and strong response to Poly I:C, as well as quantitative changes in CD8 + effector cells. Thus, transcriptomic and cell quantitative changes are associated with immune cell heterogeneity in this model to evaluate interferon responses in severe asthma.

**Supplementary Information:**

The online version contains supplementary material available at 10.1186/s12931-021-01709-9.

## Background

Asthma endotypes, disease subtypes defined by a molecular mechanism or a treatment response, have been associated with cell abundance, including eosinophils [1–3]. However, the cellular heterogeneity in immune cells within and between patients with asthma is not only restricted to absolute cell counts [3] but also includes distinct transcriptional changes in the airway [4–6] and blood [7–9]. Bulk genome-wide transcriptomics, derived from cell mixtures, support the presence of dysregulated gene expression in asthma [6, 8, 10, 11].

A limitation of bulk RNA analysis is that averaging transcriptomes can result in the loss of cell-specific signals underlying disease endotypes. Furthermore, innate and adaptive immune cellular responses are involved in asthma pathogenesis, and specific disease phenotypes result from the interaction of immune cells with the environment [12]. Among environmental influences, increased susceptibility to viral infections is strongly associated with asthma, with several studies showing impairment in different components of the antiviral response [9, 13–19]. Although these studies have informed our understanding of asthma pathogenesis, we lack a comprehensive picture of functional and quantitative responses at the single-cell level in asthma, limiting our understanding of distinct cell subtypes' contribution to specific disease endotypes.

Based on these observations, we hypothesized that single-cell analyses of a model using peripheral blood mononuclear cells (PBMCs) in asthma would reveal an impaired response to interferon (IFN). We used two complementary single-cell methods to study transcriptomic and quantitative changes in a model using PBMCs of patients with severe asthma (SA) before and after stimulation with polyinosine–polycytidylic acid (poly I:C). Poly I:C, a synthetic double-stranded RNA (dsRNA) analog and TLR3 agonist, leads to the production of type I interferons (IFNs) [20], and we compared these profiles with unstimulated PBMCs. We used DropSeq for single-cell RNA sequencing (scRNA-seq) [21] and mass cytometry (CyTOF) [22] to profile single cells in PBMCs of patients with SA and healthy controls (HC), to determine whether cell-specific signatures are associated with a decreased response to IFN.

## Methods

### Sample collection

**Fig. 1:**
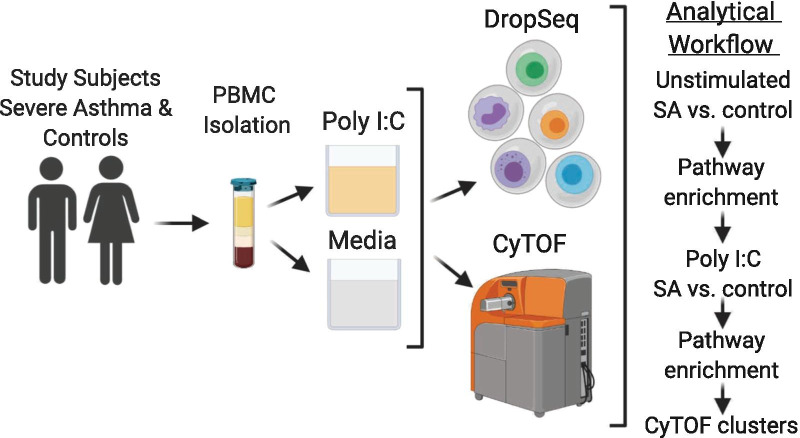
Study workflow

Human donors were recruited as part of the GenEx study at the Yale Center for Asthma and Airway Disease (YCAAD). The Yale Human Research Protection Program approved this study. Figure 1 summarizes the study's workflow.

### PBMC isolation

Whole blood was collected from 8 subjects (SA = 5; HC = 3) into BD Vacutainer® Sodium Heparin Tubes (BD Biosciences, Franklin Lakes, NJ). PBMCs were isolated using Lymphoprep and SepMate™-15(IVD) (STEMCELL Technologies, Vancouver, Canada) according to the manufacturer’s protocol. Isolated PBMCs were aliquoted and stored in cryotube vials (Thermo Scientific, Waltham, MA) with freezing medium (80% FBS, 20% DMSO) at a concentration of 5 × 10^6^ cells/mL. Cells were stored overnight in Nalgene™ Cryo 1 °C Freezing Container (Thermo Fisher, Rochester, NY) at − 80 °C, then transferred into liquid nitrogen for long-term storage. Figure 1 (created with Biorender.com) summarizes the study’s experimental and analytical workflow.

### Poly I:C preparation

Poly I:C (InvivoGen, San Diego, CA) was reconstituted to 1 mg/mL stock solution per the manufacturer's protocol. Briefly, 10 mL of endotoxin-free physiological water was added into 10 mg poly I:C. The mixture was heated up to 70 °C for 10 min, then was cooled down at room temperature for 1 h. Poly I:C stock solutions were aliquoted into 100 µL each tube and stored at − 20 °C. 500 mL cell culture medium, which was composed of RPMI 1640 media (Gibco Laboratories, Gaithersburg, MD) supplemented with 10% heat-inactivated fetal bovine serum (MilliporeSigma, Burlington, MA) and 5% Penicillin–Streptomycin (MilliporeSigma, Burlington, MA), was prepared and aliquoted before the experiment started to ensure all the following experiments were using the same medium. A working solution at 10 µg/mL poly I:C was prepared using cell culture medium 20 min before each use.

### In vitro exposure

One vial of frozen PBMCs (approximately 3 million cells) was removed from liquid nitrogen and quickly thawed in a 37 °C water bath. Cells were then transferred into 10 mL pre-warmed cell culture medium and centrifuged at 400*g* for 10 min to wash away DMSO. The supernatant was discarded after centrifugation. The cell pellet was resuspended with 3 mL 1 × BD Pharm Lyse™ (BD Biosciences, Franklin Lakes, NJ) to remove red blood cells, incubated at room temperature for 3 min, then quenched with 7 mL PBS. An aliquot of cells was removed for cell counting before centrifuging at 400*g* for 10 min. After centrifugation was completed, the supernatant was removed. Cells were resuspended with medium pre-warmed to achieve a concentration of 2 × 10^6^ cells/mL.

Cells were then plated into 96-well plates at 100 µL/well and incubated at 37 °C, 5% CO_2_ 100% humidity for 30 min. After incubation, cells were quenched with either 100 µL cell culture medium or 10 µg/mL poly I:C working solutions. The final concentrations were plain medium and 5 µg/mL poly I:C. Cells were cultured at 37 °C, 5% CO_2_ 100% humidity for 24 h.

### Drop-seq experiment

After 24 h of incubation, cells were harvested into Eppendorf tubes and centrifuged for 10 min at 400*g*. Cells were washed once with 1 mL PBS with 0.01% BSA (PBS-BSA) to remove remaining FBS, then centrifuged for 8 min at 400*g*. After discarding the supernatant, cells were then resuspended with 1 mL plain PBS. An aliquot of cells was removed for cell counting and viability recording with a hemocytometer using the trypan blue exclusion method to select samples with cell viability greater than 90%. Cells were adjusted to 140–160 live cells/µL using PBS-BSA as Drop-Seq input. The Drop-Seq experiment was carried out with the version 3.1 protocol from the McCarroll Lab [21]. Briefly, in the microfluidic channel, the barcoded beads (Chem Genes, Wilmington, MA) encounter cells, and the oil flow separates each bead-cell into droplets. Once the cells were lysed within the droplets, released mRNAs were captured by bead barcoded sequences. In practice, beads (at 120 beads/µL), cells, and oil were pumped into a microfluidic system at speeds of 3,500 µL/hr, 3,500 µl/hr, and 13,125 µL/hr, respectively. Outflow droplets were collected in 50 mL Falcon tubes. After removal of excess oil from the bottom, outflow droplets were broken by adding 30 mL 6 × Saline-Sodium citrate buffer (SSC) (MilliporeSigma, Burlington, MA) and 1 mL of 1H,1H,2H,2H-Perfluoro-1-octanol (PFO) (MilliporeSigma, Burlington, MA) followed by forceful handshakes. Ambient RNA and excess oil were washed away by an additional 6 × SSC buffer. Reverse transcription was conducted in the Eppendorf tubes. After reverse transcription, beads were washed with TE-SDS (10 mM Tris pH 8.0 + 1 mM EDTA + 0.5% SDS) buffer and stored in TE-TW (10 mM Tris pH 8.0 + 1 mM EDTA + 0.01% Tween-20) buffer at 4 °C before further steps.

Exonuclease treatment and PCR amplification were conducted within one week of reverse transcription of each sample. Beads were washed with water and then counted for PCR amplification. An apportion of 2,000 beads were put into one reaction; this would yield roughly 100 single-cell transcriptome attached to microparticles (STAMPs). Multiple PCR batches from one sample were pooled before purification to ensure that at least 600 cells per sample were included. Purified PCR products were quantified using a high sensitivity bioanalyzer (Agilent 2100 expert High Sensitivity DNA Assay, Santa Clara, CA). cDNA library was constructed using 600 pg of each sample followed Drop-Seq customized tagmentation steps. Libraries were purified using the Agencourt AMPure XP system (Beckman Coulter, Brea, CA) before sequencing. RNA sequencing was conducted using Illumina Hi-Seq 2000 instrument in pair-end 75 bp. Control and poly I:C samples for each subject were pooled into one lane for sequencing at the Yale Center for Genome Analysis.

### CyTOF

One vial of frozen PBMC from human donors was cultured with poly I:C and cell culture medium described above under in vitro exposure. Cultured cells were collected and transferred to 10 mL of pre-warmed DMEM (Gibco Laboratories, Gaithersburg, MD). Cells were centrifuged at 1700 rpm for 7 min, resuspended in 1.5 mL of cell staining buffer (CSB) (Fluidigm, South San Francisco, CA), and 1–3 million cells were transferred to an Eppendorf tube for subsequent staining. Cells were then centrifuged at 1700 rpm for 7 min, resuspended in 45 μL of CSB + 5 μL of Fc blocker (Biolegend, San Diego, CA), and incubated at room temperature for 10 min. Afterward, 50 μL of freshly prepared antibody cocktail was added (total staining volume 100 μL) and incubated on ice for 30 min. A summary of the antibodies used can be found in Additional file 1: Table S1. Next, cells were washed with CSB and stained for cell viability with 7.5 μM Cisplatin (Fluidigm, South San Francisco, CA) in RPMI 1,640 media for 1 min and quenched with pure fetal bovine serum (MilliporeSigma, Burlington, MA). Cells were then washed with CSB, fixed, and permeabilized with the eBioscience Foxp3 Transcription Factor Staining Buffer Set (3:1 dilution) (Thermofisher Scientific, Waltham, MA) and stained with DNA intercalator (125 nM Iridium-191/193) overnight at 4 ºC. The next day, cells were washed first with CBS and then with ddH2O water (centrifuge speed 2100 rpm for 10 min). Cells were then diluted to a 1 × 10^6^ cells/mL concentration with a 1:7 dilution of beads solution (Fluidigm, South San Francisco, CA). Lastly, cells were filtered into a 35 μm cell strainer cap tube and were acquired at a rate of 300–500 cells/second using a CyToF2 mass cytometer (Fluidigm, South San Francisco, CA) at the Yale CyTOF facility.

### Data analysis

Dropseq-derived sequencing reads were aligned to Genome Reference Consortium Human Build 38 (GRCh38) and then binned onto the cell barcodes corresponding to individual beads using Drop-Seq tools [23]. We filtered out cells containing less than 200 genes and genes not detected in at least three cells to ensure qualifying genes and cells. Library-size normalization was performed with a global scaling method by Seurat v 3.1.1 [24]; briefly, the UMI-collapsed gene expression values for each cell barcode were scaled by the total number of transcripts and multiplied by 10,000. Data was then natural log-transformed before any further downstream analysis.

The number of input reads ranged between 30.7 × 10^6^ and 84.8 × 10^6^. The number of uniquely mapped reads ranged between 42 and 79%, with no differences between groups (p = 0.63 and p = 0.32, respectively) (Additional file 1: Table S2).

A mitochondrial gene expression threshold of 5% was used to remove low-quality/dying cells that tend to show mitochondrial contamination. Highly variable genes were detected and calculated based on average expression and dispersion for each gene. Genes with high variability (n = 2,000) were used before scaling the gene expression data (Additional file 1: Table S3). A regression analysis using scaled data was performed using RNA counts, percentages of mitochondrial gene expression, and batch to remove unwanted variation sources. An independent analysis using cell cycle scoring of scaled data did not show any particular bias toward a cell cycle-specific phase. Consequently, cell cycle variation was not included in the regression analysis. The Elbow plot and the JackStraw procedures were used to identify principal components (PCs) and yielded similar results.

Principal components analysis (PCA) was performed based on these highly variable genes. Statistically significant PCs were selected for clustering analysis. The Uniform Manifold Approximation and Projection (UMAP) dimensional reduction technique was used to visualize the dataset. The positive and negative markers for each cluster were found through differential expression analysis, and only genes with expression percentage above 20% were retained. These markers were used to denote cell identities. Differential gene expression was determined using the likelihood-ratio test for single-cell feature expression [25], using a fold change threshold of 10%. To identify changes between stimulated and unstimulated cells, we implemented the integration method described by Stuart and Butler et al. [26]. P values were adjusted using Bonferroni correction, and only p < 0.05 were reported and used in downstream analyses.

PHATE [27] embedding of CD4 + T cells was used to generate a pseudotemporal reconstruction of branching lineages [28]. Pseudotime reconstruction was followed by SCENIC analysis [29] to identify single-cell regulons. Briefly, a regulon is a set of genes in which a specific regulatory gene controls their expression. Predicted regulon activities per cell were calculated using the pySCENIC package with default settings. To this end, the cisTarget databases and the transcription factor motif annotation were used [30]. The list of human transcription factors was obtained from the Aerts Lab website [31].

Pathway maps, gene ontology (GO) processes, process networks, enrichment by diseases, and network analysis were performed with MetaCore version 20.1 build 70,000 (Clarivate Analytics, Philadelphia, PA), using differential gene expression outputs obtained from Seurat and SCENIC analyses.

CyTOF fcs files were normalized with fca_readfcs [32], (retrieved November 6, 2019). Following normalization, fcs files were processed further using Cytobank v.7.3.0, where gating was performed using DNA, event length, cisplatin, and CD45, to identify single live immune cells. Gated fcs files were exported and analyzed further using the cytofkit R package (v 1.12.0) [33]. Clustering was performed in cytofkit using the Rphenograph method with the CD45, CD19, CD1c, CD4, CD8a, CD16, CD123, CXCR3, CD14, CD127, CCR6, CD25, CD3, CD38, HLA-DR, and CD56 markers. CytofAsinh was selected as the data transformation method, t-distributed stochastic neighbor embedding (tSNE) was used as the dimensionality reduction method, and k was set at 30 to analyze 160,000 cells (10,000 cells per sample; SA = 10, HC = 6). Non-specific staining was seen in CRTH2 and CLEC9A; consequently, these markers were not used for clustering and did not affect cell type identification.

All analyses were performed with R software version 3.5.1. Values are reported as means, standard deviation, percentages. The Chi-square test was used in categorical data comparisons; the t-test was used in continuous data and the two-sample test for equality of proportions with continuity correction for proportions. All p-values were adjusted by false discovery rate (FDR) or Bonferroni methods unless indicated otherwise. P values < 0.05 were considered significant.

The datasets generated and/or analysed during the current study are available in the Sequence Read Archive (SRA) repository (pending number).

## Results

### Subject characteristics

Subjects with SA (n = 5) and HC (n = 3) had similar age and sex distribution (Table 1). Subjects with SA had higher BMI (p = 0.04). Only a minority of study subjects had cigarette exposure history, SA (n = 2) and HC (n = 0), and the two former smokers (SA) had one pack-year history of cigarette smoking each; neither was actively smoking. All subjects with SA were using a combination of inhaled corticosteroids and long-acting β_2_ -agonists. Additional therapies included montelukast, tiotropium, and omalizumab. Despite lower mean and median pulmonary function values in SA, there were no statistical differences compared to HC. All subjects with SA fulfilled the SA definition by ATS/ERS guidelines [34]. Four patients were using high doses of inhaled corticosteroids, and one patient was being treated with omalizumab. Two patients were classified as atopic asthma, three as eosinophilic asthma (two with absolute blood eosinophil count > 300 cells/microliter, one with sputum eosinophils 2% and < 40% neutrophils). Only one patient was defined as a frequent exacerbator, with two exacerbations in the previous year. These patients did not have frequent viral exacerbations in the year before the study visit. The last recorded exacerbation in all patients was more than three weeks before the study. No patient was using systemic corticosteroids at the time of blood collection.

**Table 1:** Subject Demographics

	Severe asthma (n = 5)	Healthy control (n = 3)
Age (years)	43 ± 17	38 ± 15
Sex (Female: Male)	4:1	2:1
Race (n)		
Caucasian	3	1
African-American	0	1
Asian	-	1
Other	2	0
Hispanic (n)	3	1
BMI (Kg/m^2^)	36 ± 6	23 ± 4
Smoking (n)	2	0
Pack years	1 each	-
Therapies % (n)		
Inhaled corticosteroids	100 (5)	-
Long-acting β_2_-agonist	100 (5)	-
Montelukast	60 (3)	-
Tiotropium	40 (2)	-
Omalizumab	20 (1)	-
FEV_1_ pre-bronchodilator (% Predicted)	80 ± 18	91 ± 7
FEV_1_/FVC	78 ± 5	82 ± 14
FeNO (ppb)	23 ± 14	23 ± 16
Absolute blood eosinophils (cells/microliter)	260 ± 196	6 ± 11

*BMI* body mass index, *FEV*_*1*_ forced expiratory volume 1s, *FVC* forced vital capacity, *FeNO* fractional exhaled nitric oxide

**Table 2:** CyTOF cluster percentages by condition

Cell type	Unstimulated		Poly I:C	
	HC (n = 30,000)	SA (n = 50,000)	HC (n = 30,000)	SA (n = 50,000)
B cells	17.4	8.3	14.3	9.1
CD4 + T cells	21.2	15.1	22.6	18.5
CD4 + memory	1.9	1.1	1.8	1.3
Th1 cells	7.1	11.2	6.8	9.1
TH17 cells	4.3	3.1	3.3	2.7
CD8 + T cells	12.7	22.9	16.4	24.8
CD8 + effector	3.9	10.7	4.3	7.7
Monocyte 1	5.7	4.5	4.7	3.0
Monocyte 2	3.5	4.0	4.8	4.7
Natural killer cells	6.0	1.9	3.9	1.6
Plasmacytoid dendritic cells	0.1	0.1	0.2	0.1

*HC* healthy control, *SA* severe asthma

*Remaining percentages were associated with technical artifacts

### Single-cell RNAseq analysis of PBMCs

**Fig. 2:**
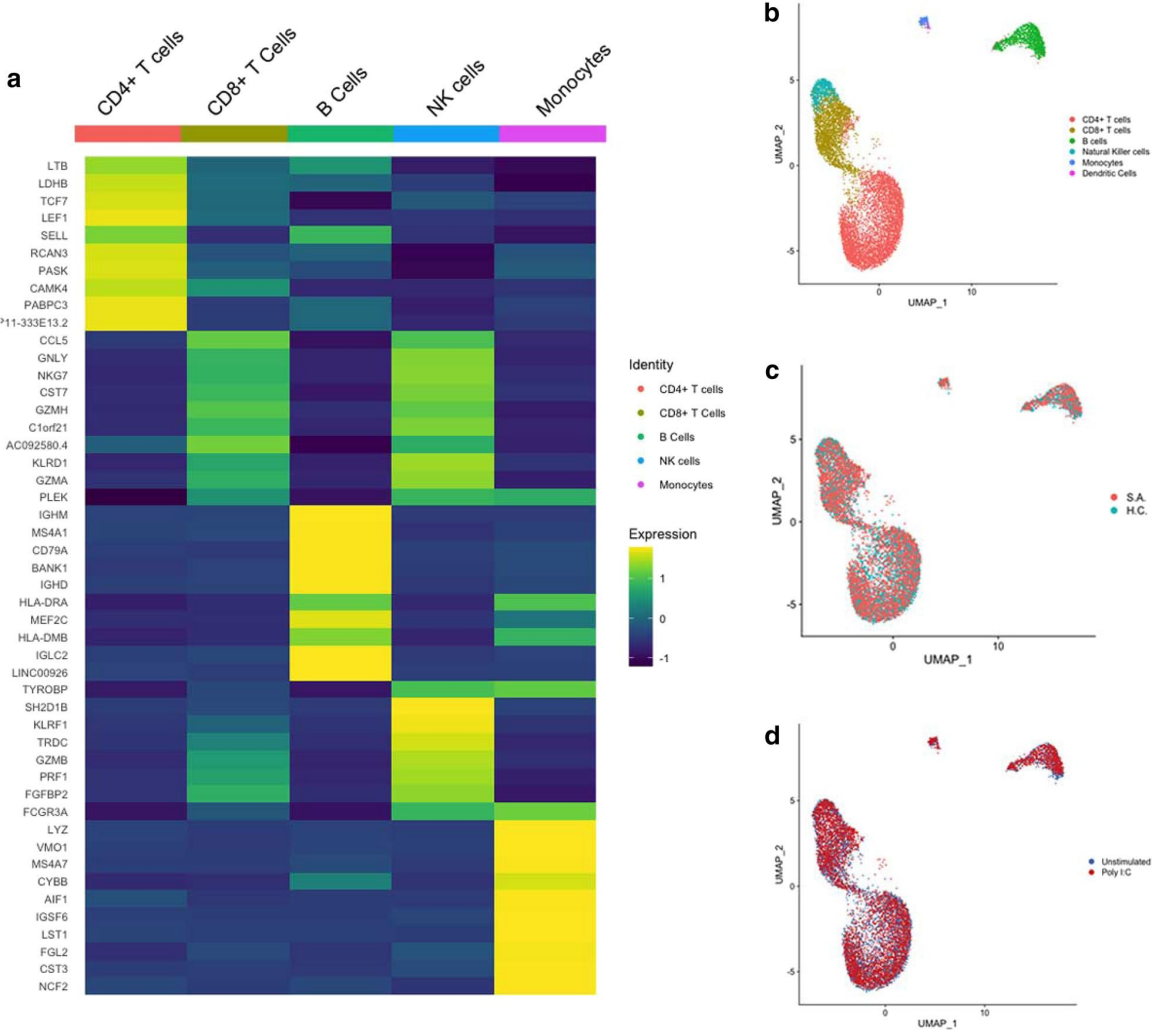
Single-cell RNAseq of PMBCs Identifies Distinct Clusters of Cells. **a** Top ten cluster markers for the five main cell types in all cells (n = 9390). **b** UMAP of all cell clusters including dendritic cells (n = 9414). **c** UMAP of all cell clusters by disease status, severe asthma (SA) (n = 6099) and healthy control (HC) (n = 3315). This figure demonstrates a similar distribution of cells across patients with severe asthma and healthy controls. **d** UMAP of all cell clusters by stimulation status, unstimulated (n = 4283) and poly I:C (n = 5131). This figure demonstrates a similar distribution of unstimulated and poly I:C stimulated cells

To investigate transcriptional changes at the single-cell level in unstimulated cells and response to poly I:C stimulation, we isolated PBMCs from patients with SA and HC. Isolated PBMCs were exposed to poly I:C (stimulated) or plain culture media (unstimulated) for 24 h before single-cell isolation using the DropSeq method [21]. Figure 1 summarizes the study’s single-cell transcriptome attached to microparticles (STAMPs)workflow. Similar numbers of STAMPs were retrieved per condition (unstimulated: 600 ± 0; poly I:C: 500 ± 295, p = 0.34). The number of input reads ranged between 30.7 × 10^6^ and 84.8 × 10^6^. The number of uniquely mapped reads ranged between 42 and 79%, with no differences between groups (p = 0.63 and p = 0.32, respectively) (Additional file 1: Table S2). After filtering by mitochondrial gene expression, 9,584 cells were used in downstream analyses. Fifteen principal components (PCs) were used to identify correlated gene sets to define cell clusters (Fig. 2a).

Specific cell markers were used to classify distinct groups of cells using singleR [35]. Further manual curation complemented the singleR assignment. Total cell count per condition was: 4,283 unstimulated and 5,131 poly I:C stimulated cells. The number of cells per disease status was: 6,099 for subjects with SA and 3,315 for HC. In both SA and HC subjects, CD4 positive T cells were the predominant group of cells, 54% in both, while dendritic cells (DCs) were the smallest group and ranged from 0.2 to 0.4%, respectively. Given the absence of enough DCs in SA subgroups (1 cell in unstimulated, 3 cells in poly I:C), we could not perform analyses across all conditions. Consequently, we concentrated our analyses on 9,390 cells, including CD4 + T cells, CD8 + T cells, natural killer (NK) cells, B cells, and monocytes. Genes associated with specific cell identity are summarized in Additional file 1: Table S4. The heatmap in Fig. 2a illustrates the top ten representative cell cluster biomarker genes (cell-specific transcriptional programs in PBMCs).

### Unstimulated single cells of severe asthmatics display a pro-inflammatory state

**Fig. 3:**
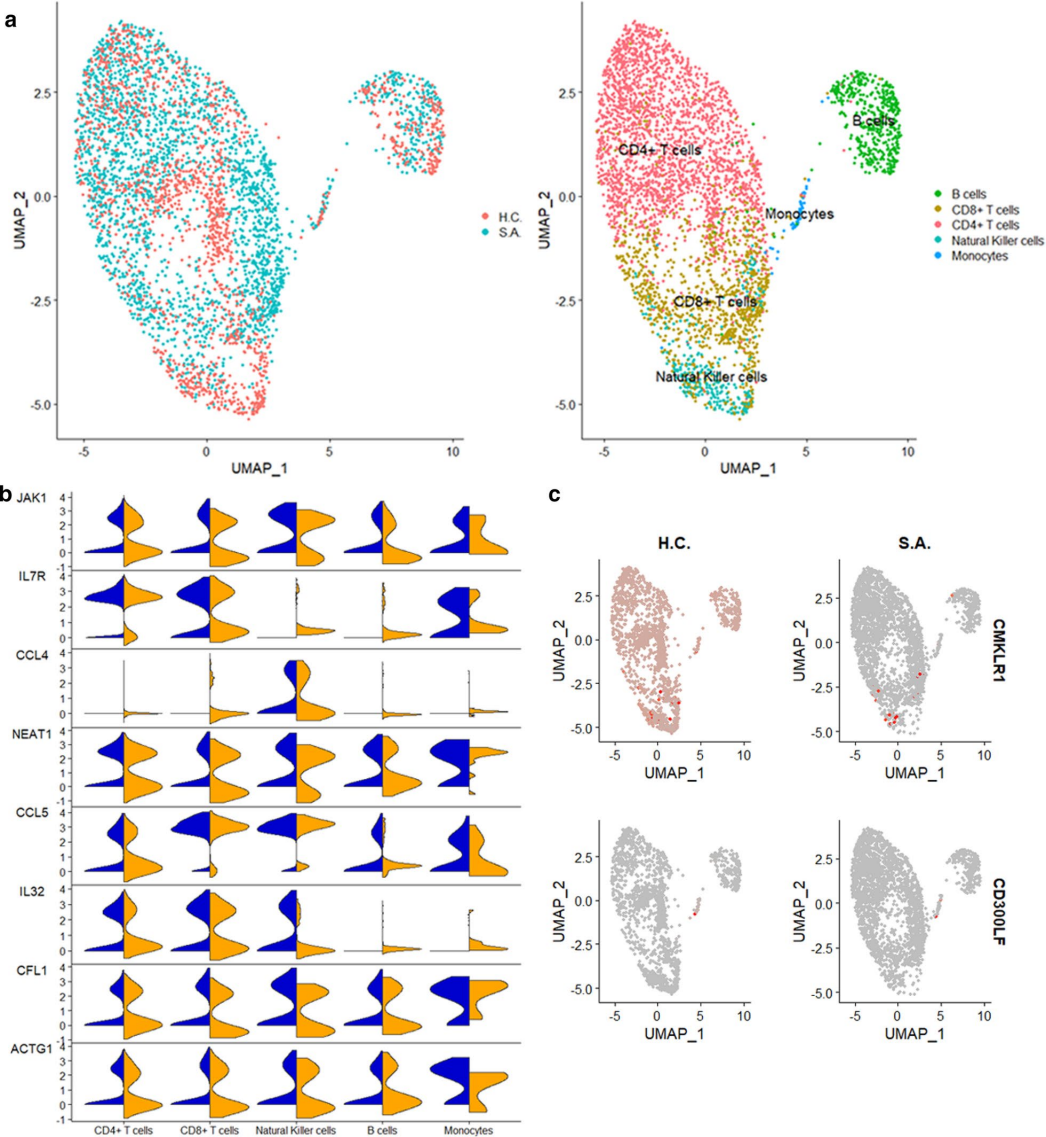
Single-cell RNAseq of unstimulated cells. **a** UMAP of unstimulated PBMCs demonstrates a similar distribution of cells between severe asthma (SA) and healthy controls (HC). **b** Several pro-inflammatory transcripts including *JAK1, IL7R, CCL4, NEAT1, CCL5, IL32, CFL1*, and *ACTG1* are highly expressed in cells from patients with SA (blue) compared to HC (orange). **c** Expression of the anti-inflammatory transcripts *CMKLR1* and *CD300LF* were lower in SA than HC

To identify gene-expression differences between SA and HC in each specific cell subset in unstimulated cells, we compared the PBMCs of subjects with SA and HC. Unstimulated cells in SA and HC had overlapping distribution (Fig. 3a). Transcriptional differences between unstimulated PBMCs are summarized in Additional file 1: Table S5. Among the most representative transcripts, the Janus kinase (*JAK1*) was highly expressed in CD4 + T cells, CD8 + T cells, NK cells, B cells, and monocytes of SA compared to HC. CD4 + T, CD8 + T, NK, and B cells of SA also showed high expression of the long non-coding RNA (lncRNA) *NEAT1*, associated with Th2 differentiation [36] (Fig. 3b). In SA, the pro-inflammatory cytokine *IL32* was also highly expressed in all cell types except monocytes (Fig. 3b).

### Severe asthmatics demonstrated cell-specific heterogeneity of type 2 inflammatory transcript expression at baseline

CD4 + T and B cells and monocytes of SA displayed higher expression of *CCL5*, a known eosinophil chemoattractant and an activator [37]. In comparison, CD8 + T cells of SA had a higher expression of *CCL4* (MIP-1-beta). In monocytes of SA, the chemerin chemokine-like receptor 1 (*CMKLR1*), the receptor for the chemoattractant adipokine chemerin/RARRES2 and the omega-3 fatty acid-derived molecule resolvin E1 [38], and *CD300LF*, a receptor involved in the negative regulation of mast cell activation [39], were both downregulated. However, in NK cells of SA (Fig. 3c), *CMKLR1* was upregulated, demonstrating cellular heterogeneity in this receptor's expression.

### Pathway enrichment of scRNA-seq transcriptomes identified enhanced cell adhesion and signaling in severe asthmatics at baseline

To determine which pathways were enriched in unstimulated cells from SA, we performed enrichment analyses by cell type (Additional file 1: Table S6). This analysis demonstrated the enrichment of pathways involved in cytoskeletal remodeling and cell adhesion in CD4 + T, CD8 + T, NK, and B cells from SA (FDR p < 0.01). The *CCR3* signaling pathway was also enriched in CD4 + T, CD8 + T, NK, and B cells from SA (FDR p < 0.01). A network of genes involved in NK cell cytotoxicity was also highly expressed in SA (FDR p < 0.01). NK cells from SA also demonstrated lower expression of multiple genes involved in phagocytosis and antigen presentation (FDR p < 0.01). Enrichment of B cells showed that B cells of SA were enriched for *CXCR4* signaling (FDR p < 0.01). Thus, CD4 + T, CD8 + T, NK, and B cells of SA demonstrated similar pro-inflammatory changes.

### Pathway enrichment identified monocyte-specific inflammatory modules in severe asthmatics at baseline

In contrast with other cell types, monocytes of SA demonstrated enrichment for a Th17 pathway in asthma (FDR p < 0.01), and the main gene ontology (GO) processes were regulation of immune system process and cellular response to cytokine stimulus (FDR p < 0.01). Also, in contrast with CD4 + T, CD8 + T, NK, and B cells, monocytes of SA showed decreased expression of *CFL1* and *PFN1*, involved in the CCR3 signaling pathway. Across all unstimulated cell types of SA, monocytes demonstrated unique pathway enrichment patterns. Single genes and pathways with pro-inflammatory effects were highly expressed in patients with SA and decreased expression of genes involved in resolution or negative regulation of inflammation. Together, these transcriptional changes are consistent with a pro-inflammatory status in unstimulated single cells of SA.

### PBMC responses to Poly I:C stimulation demonstrated induction of IFN pathways in severe asthma

We hypothesized that in this model, PBMCs of SA might have decreased interferon-stimulated genes (ISGs) expression following exposure to poly I:C. We performed single-cell RNAseq analysis of poly I:C stimulated PBMCs from SA and HC. In this experiment, interferon gamma (*IFNG*) was the only interferon expressed in PBMCs and its expression was only present in 23 cells (Additional file 2: Figure S1). Contrary to our hypothesis, poly I:C stimulation was associated with higher expression of ISGs in SA (Additional file 1: Table S7). Several ISGs and genes involved in interferon pathways shared the same behavior across cell types in SA, except for *BIRC3, EDN1, FAM65B, IFITM2, ISG20*, and *SELL*, which were downregulated in B cells (Additional file 1: Table S7). The overall response across cell types in SA was associated with higher expression of multiple interferon pathway genes than HC.

### Th1 and Th2 transcriptomic networks were concomitantly and broadly expressed following Poly I:C stimulation

The plant homeodomain finger protein 11 (*PHF11*), a positive regulator of Th1 cytokine gene expression [40], was highly expressed in monocytes, CD4 + T, CD8 + T, and NK cells of SA, despite the concomitant increased expression of the lnc *NEAT1* associated with Th2 differentiation [36]. *IL7R*, part of the heterodimeric receptor for the thymic stromal lymphopoietin (TSLP), and *JAK1* were highly expressed in CD4 + T, CD8 + T, and B cells from SA. Together, these findings suggest that multiple cells from SA have a robust induction of genes involved in both Th1 and Th2 inflammation.

### Pathway enrichment of Poly I:C stimulated cells demonstrated IFN signaling induction with concomitant downregulation of antigen presentation in severe asthmatics

We performed enrichment analyses in differentially expressed genes between SA and HC following poly I:C stimulation (Additional file 1: Table S8). In all cell types of SA, immune pathways of IFN-alpha/beta signaling via *JAK/STAT* and/or IFN-alpha/beta signaling via mitogen-activated protein kinases (MAPKs) were enriched, unlike in HC. CD4 + cells of SA also had increased expression of genes involved in the generation of memory CD4 + T cells, including *TRAC* and *TRBC2,* and *JAK1*. In CD8 + T and B cells of SA, a pathway involved in negative regulation of HIF1A function was enriched for highly expressed genes in SA (FDR p < 0.01). Enrichment by disease status of downregulated genes in CD8 + T cells of SA identified several genes involved in chronic obstructive pulmonary disease (FDR p < 0.01). Furthermore, in monocytes of SA, genes involved in antigen presentation by MHC class I were downregulated, unlike in monocytes of HC (FDR p < 0.01). Thus, single cells from SA demonstrated robust induction of IFN signaling pathways and induction of memory in CD4 + T cells, with downregulation of pathways involved in antigen presentation.

### Poly I:C stimulation identified antigen presentation heterogeneity in PBMCs from severe asthmatics

**Fig. 4:**
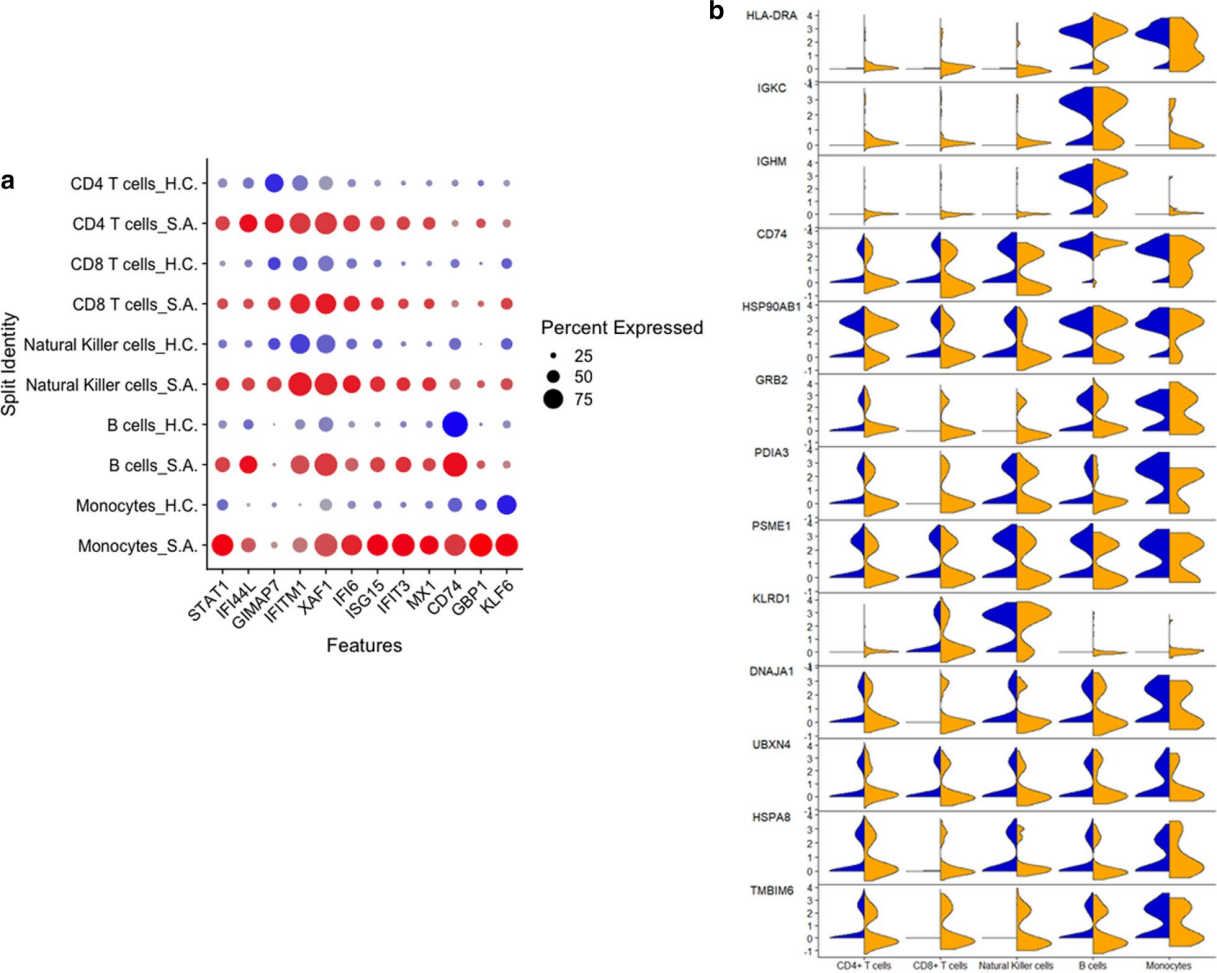
Single-cell RNAseq of PBMCs stimulated with poly I:C. **a** Poly I:C stimulation led to a robust increase in the expression of STAT1 and multiple interferon-stimulated genes in SA. **b** Genes involved in antigen presentation, including HLA-DRA, IGKC, IGHM, and CD74 had a heterogeneous expression across cell types and between SA and HC. Similarly, genes involved in ubiquitination and unfolded protein response also demonstrated a heterogeneous response across cells and disease status

Given the decreased expression of antigen presentation genes in monocytes, we focused on cell-specific changes in phagocytosis and antigen presentation pathways compared to other cells. We found that CD4 + T cells demonstrated enrichment of antigen presentation genes; however, elements of antigen presentation pathways and phagocytosis were differentially expressed across cell types and disease status, with *HLA-DRA, IGKC*, and *IGHM* demonstrating low expression in cells from SA. In contrast, other genes, including *CD74, HSP90AB1, PDIA3*, and *PSME1*, were highly expressed in SA (Fig. 4b). Therefore, genes involved in antigen presentation and phagocytosis had distinct expression patterns in SA in response to poly I:C stimulation. Similarly, several genes involved in the unfolded protein response and ubiquitination showed a heterogeneous expression pattern in cells of SA (Fig. 4b). Together, these findings identify cell-specific differences in antigen presentation, phagocytosis, unfolded protein response, and ubiquitination pathways in SA cells.

### Trajectory analysis of CD4 + T cells identified five main regulons in response to poly I:C stimulation

**Fig. 5:**
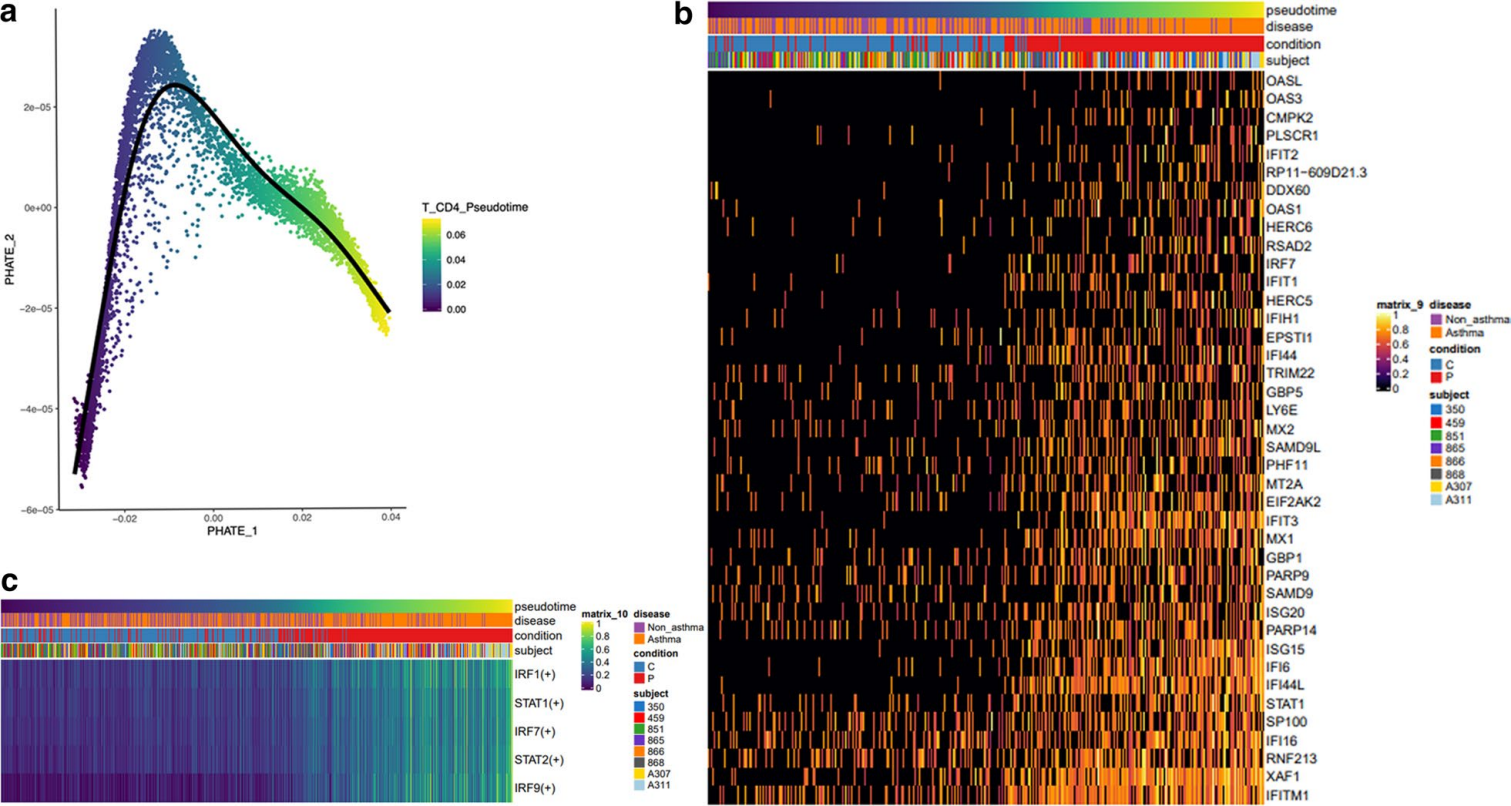
Pseudotime analysis of CD4 + T cells demonstrated a strong association with interferon signaling in severe asthma. **a** PHATE analysis for pseudotemporal reconstruction of CD4 + T cells. **b** Heatmap of transcripts correlated with pseudotime identified multiple interferon-stimulated genes correlated with the response to poly I:C. **c** Regulon analysis identified *IRF1, STAT1, IRF7, STAT2*, and *IRF9* as the top five transcription factors positively correlated with pseudotime and critical regulators of positively correlated genes in response to poly I:C

To better understand IFN signaling pathways in response to poly I:C, we next examined the main transcription factors associated with poly I:C stimulation in CD4 + T cells of SA and HC using pseudotime analysis with PHATE [27]. PHATE embedding of CD4 + T cells was used to generate a pseudotemporal reconstruction of branching lineages [28] and identify the trajectory, as seen in Fig. 5a. The trajectory inference was then correlated with CD4 + T single-cell transcripts to identify co-expressed genes and transcription factors correlated with pseudotime [29]. This method identified 456 genes correlated with pseudotime at Bonferroni p < 0.05 (Additional file 1: Table S9). Positively correlated genes were enriched for interferon type I signaling via JAK/STAT (FDR p < 0.01) (Additional file 1: Table S10), consistent with the pathway enrichment in CD4 + T cells of SA (Additional file 1: Table S8). In contrast, negatively correlated genes were enriched for the signal recognition particle (SRP)-dependent protein targeting the membrane during translation (FDR p < 0.01) (Additional file 1: Table S10). Regulon analyses found a strong correlation between these genes and five transcription factors, *IRF1*, *STAT1*, *IRF7*, *STAT2*, and *IRF9 (*Bonferroni p < 0.05) (Fig. 5c; Additional file 1: Table S11). These transcriptional and master regulatory responses were more prominent in SA cells than in HC (Figs. 5b, c). These observations suggest that antiviral transcripts in PBMCs from SA are regulated by five main transcription factors, *IRF1*, *STAT1*, *IRF7*, *STAT2*, and *IRF9.*

### CyTOF analysis identified cell abundance differences in severe asthma

**Fig. 6:**
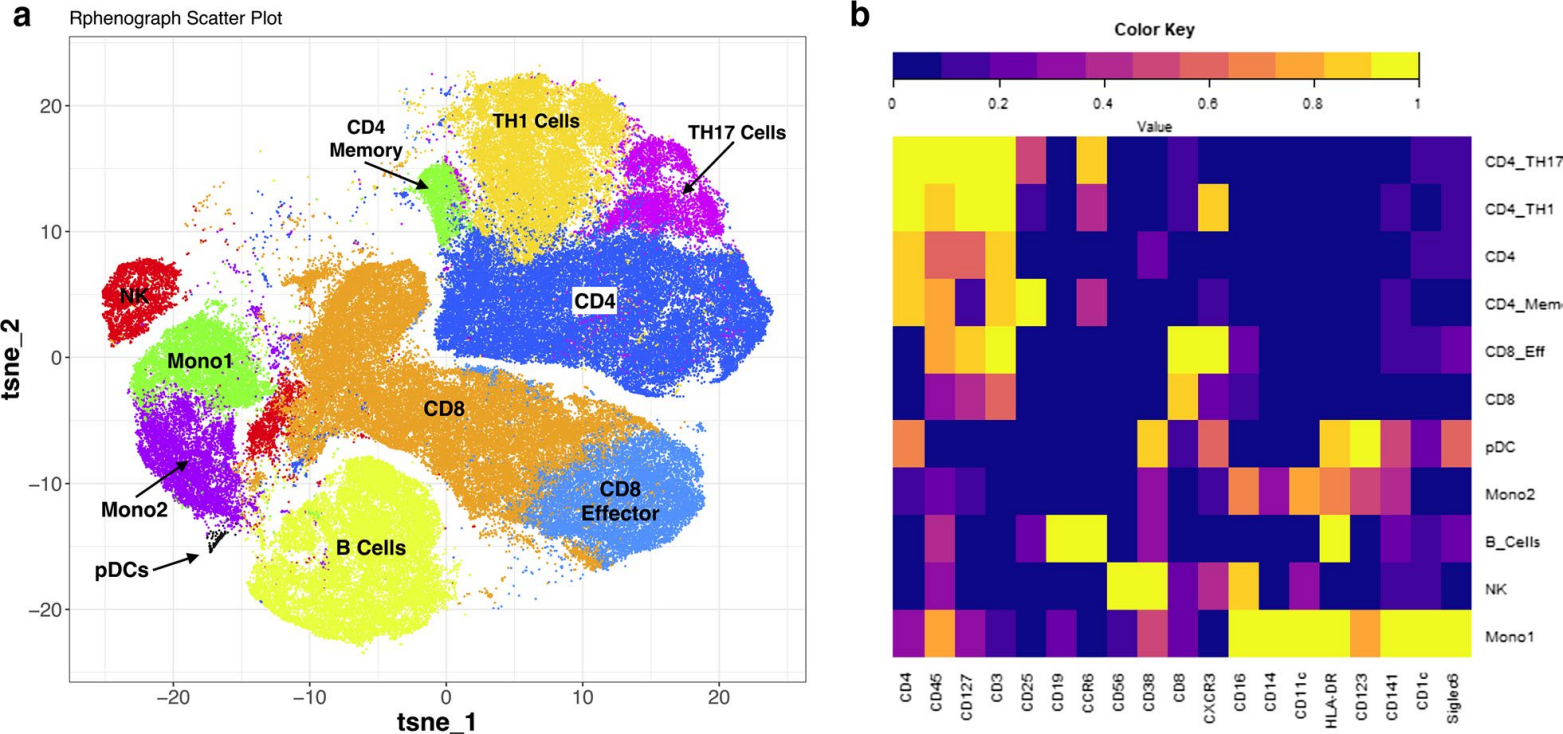
CyTOF analysis of PBMCs from the same subjects profiled with single-cell RNAseq identifies a similar distribution of cells. **a** tSNE plot of cell clusters on CyTOF. **b** Heatmap of cell surface markers and clusters determined by CyTOF. This is a simplified version of all the clustering results; the Additional file 2: Figure S2 includes all clusters. CD4 cells, together with CD4 effector cells (CD4 Eff), CD4 memory (CD4 Mem), CD4-Th1, and CD4-Th17 cells, are clustered independently from B cells, natural killer (NK), dendritic cells (DCs). CD8 cells are also clustered independently and include CD8 effector (CD8 Eff), CD8 central memory (CD8 Tcm), CD8 naïve and memory (CD8 N&M), CD8-low cells, and CD8 cells. A small subset of Cytokine-induced killer cells (CIK) was identified in this analysis

To identify and quantify PBMC subpopulations at baseline and following stimulation with poly I:C, we performed CyTOF staining of 21 immune cell surface markers (Additional file 1: Table S1). Clustering of 160,000 cells in 12 samples from the same subjects profiled with scRNA-seq (SA = 5; HC = 3) identified 28 cell clusters (Additional file 2: Figure S1 and Table 2). Some clusters were formed by technical artifacts consistent with double staining clusters 3, 6, 7, 11, 12, 13, 15, 17, 22, 25 and 26 and decreased staining (clusters 21). The presence of these clusters did not affect the identification of main cell subsets and there were no differences between conditions or disease status. Figure 6 is a simplification of the CyTOF clusters from the Additional file 2: Figure S2.

Comparison between CyTOF-derived SA and HC cell percentages demonstrated consistency with scRNA-seq results. CD4 + and CD8 + T cell clusters accounted for most of the cells (Table 2).. We found differences in unstimulated cell percentages between SA and HC, with higher percentages of CD8 + T cells (22.9% vs. 12.7%, p < 0.01) and CD8 + effector T cells (10.7% vs. 3.9%, p < 0.01), and lower lower percentages of B cells (8.3% vs. 17.4%, p < 0.01), and NK cells (1.9% vs. 6%, p < 0.01).

Following poly I:C stimulation, SA had a 3.5% increase in CD4 + T cells (p < 0.01), with a concomitant 3% decrease in CD8 effector cells (p < 0.01) and 2.1% in CD4 + Th1 cells (p < 0.01). In contrast with SA, HC demonstrated a 3.7% increase in CD8 + T cells (p < 0.01), with a concomitant decrease of 3% in B cells (p < 0.01), and 2.1% in NK cells (p < 0.01). Together, these findings show differences in the patterns of relative cell abundance in SA compared to HC. Specifically, higher CD8 + and CD8 + effector cells and lower B cells. Similarly, we observed differences in the cell abundance patterns by disease state in response to poly I:C.

## Discussion

In this study, we comprehensively analyzed a model to evaluate the response of PBMCs to the TLR3 agonist poly I:C using scRNA-seq and CyTOF on samples from patients with SA and HC. Contrary to our expectation, this study shows that cells of asthmatics do not have an impaired response to IFN stimulation. We found that stimulation with poly I:C of PBMCs from SA patients led to a higher expression of ISGs than in HC cells. Despite the inherent limitations of studying only five individuals with SA, this observation suggests that the interferon pathways stimulated by poly I:C do not appear to be impaired in all patients with SA. Furthermore, we identified quantitative differences in cell composition in SA using CyTOF. These results suggest that functional (mRNA) and quantitative differences (CyTOF) in PBMCs are present in SA and may underlie the observed results following stimulation with poly I:C.

We hypothesized that specific cells of SA have an impaired response to IFN, based on previous studies showing impaired antiviral immunity in asthma [13, 14, 19]. The induction of ISGs across CD4 + T, CD8 + T, NK, B cells, and monocytes of SA patients was robust and was supported by the results of pathway enrichment analyses demonstrating multiple instances of immune responses linked to IFN; specifically, IFN-alpha/beta signaling via JAK/STAT and IFN-alpha/beta signaling via MAPKs.

Several reasons may account for these differences between our findings and previous studies. First, we explored a particular subset of patients with SA with high type 2 inflammation (all with atopic or eosinophilic asthma). However, the response to poly I:C may vary across asthma endotypes, particularly in exacerbation-prone subgroups [41–43], furthermore, differences in demographic features and maintenance therapies may influence these observations. Second, we examined PBMCs, while other studies have evaluated the airway compartment [13, 14]. Therefore, our results must be interpreted in the context of an in vitro model rather than extending into a large heterogeneous population of patients with severe asthma.

A potential explanation for the enhanced response to poly I:C may be related to the observed increase in baseline expression of multiple pro-inflammatory and signaling genes, including *JAK1*, in SA. We had previously identified a baseline increase in *STAT1* expression in PBMCs of patients with recurrent infectious exacerbations [9]. *JAK1* and *STAT1* converge in an immune pathway involved in response to IFN and inflammation [44]. Although the *JAK1-STAT1* pathway may explain the differences in response to IFN seen here and in our previous study, the association between asthma and this pathway is complex. It may be affected by inhaled corticosteroids and may represent a distinct asthma endotype [45, 46]. Thus, transcriptomic changes in the *JAK1-STAT1* pathway may account for some of the results seen in response to poly I:C stimulation in our model.

We found that *STAT1* was one of the main regulators enriched in response to poly I:C. Previous studies have shown that the transcriptional role of *STAT1* in asthma appears to be associated with corticosteroid insensitivity and Th1 bias [45, 46]. Similarly, *IRF1* has been associated with corticosteroid insensitivity [47], and *IRF9* with interferon networks in the airway epithelium [48]. In contrast, *IRF7* has been associated with allergic airway inflammation by regulating type 2 innate lymphoid cells [49]. The observed increase of *JAK1* expression in unstimulated cells, combined with a prominent role for *STAT1* in steroid and poly I:C responses, suggests that the *JAK1-STAT1* pathway holds clues to the complex interaction between asthma, therapeutic response to steroids, and antiviral responses.

In addition to finding increased expression of *JAK1* in unstimulated cells of SA, we found high expression of pro-inflammatory genes including *IL32*, a cytokine with pro-inflammatory and antiviral activity [50]; genes involved in Th2 inflammation [36, 37]; and *CCL4*, involved in asthma exacerbations [51]. These changes are also accompanied by the downregulation of the *CMKLR1* receptor and *CD300LF* involved in anti-inflammatory mechanisms [38, 39]*.* Together, these single-cell transcriptomic changes indicate pro-inflammatory status at baseline in PBMCs of patients with SA.

Although our central question on the response of PBMCs to poly I:C, a synthetic IFN trigger [20], focused on antiviral pathways, we found cell-specific heterogeneity in antigen presentation, phagocytosis, and unfolded protein response in SA. Antigen presentation in asthma has multiple implications in disease pathogenesis and intercellular communication [52–54], while the unfolded protein response is dysregulated in asthma and airway inflammation [55, 56]. Thus, cell-specific changes in antigen presentation and unfolded protein response pathways may underlie disease heterogeneity in asthma.

An additional insight from our studies that may underlie asthma's heterogeneity is the quantitative difference in cell composition in SA, using CyTOF. We found differences in multiple cell population percentages between HC and patients with SA using CyTOF. Although variation in eosinophil count has led to the identification of eosinophilic asthma endotypes [3, 57, 58], the cell abundance of other immune cell populations has not been explored in detail. However, it may be associated with distinct asthma features [59]. The identification of differences in cell percentages in unstimulated cells and disease specific changes following poly I:C stimulation, particularly in CD8 + T cells and Th1 cells, combined with the specific functional changes seen in sc-RNA-seq, may amplify the effect of the functional cellular changes in response to environmental stimuli when quantitative cell changes are combined with functional changes. This observation deserves further prospective evaluation to determine whether quantitative and qualitative changes in PBMCs are responsible for increased between-patient heterogeneity.

We are aware of the limitations of studying a model using cells from a highly selected group of individuals with asthma. However, we leveraged two single-cell methods to assemble a dataset with thousands of cells representing patients at the high end of the disease severity spectrum. Although these transcriptional changes need to be studied in milder asthma, our findings inform future study design, including the need to enrich dendritic cells to power single-cell analyses in the PBMC compartment. Furthermore, despite heterogeneity in SA, we selected individuals with evidence of an increased type 2 response, allergic asthma, or eosinophilic asthma. It is unknown whether demographic features between SA and HC influenced some of the transcriptomic differences. Although, we would expect a more robust response to poly I:C in younger individuals (HC). An additional limitation is the use of frozen cells rather than freshly isolated cells; however, previous studies have demonstrated that DMSO is the preferred method for cryopreservation before scRNA-seq [60] and that transcriptional profiles are not altered by this method [61]. We sought to complement the use of single-cell RNAseq with CyTOF of cells from the same individuals to enable the interpretation of both functional and quantitative changes in this model of response to poly I:C. These two complementary methods create a detailed profile of this model based on single-cell mRNA and protein detection. Although it is not possible to extrapolate these findings to all patients with asthma, we expect to see similar, albeit less dramatic, changes in a larger asthma cohort.

Impairments in response to interferons have been associated with asthma pathogenesis [9, 13–19]. The use of two single-cell methods to analyze a model of the response of PBMCs from severe asthmatics to poly I:C, a synthetic IFN trigger, enabled the identification of robust ISG changes and quantitative changes in CD8 + and Th1 cells. The presence of increased JAK1 expression at baseline in SA may hold clues to these responses. Together, these observations provide evidence of qualitative (scRNA-seq) and quantitative (CyTOF) differences in SA that may be associated with disease pathogenesis. These findings imply that single-cell heterogeneity across patients in SA may be associated with disease heterogeneity. The combination of multi-omics assays and external perturbations, such as those reported here, enables the study of functional and quantitative immune profiles in patients with asthma to identify alterations in distinct molecular pathways and enable the identification of specific asthma endotypes. Future cohort studies using a combination of quantitative and qualitative single-cell methods in asthma cohorts can help validate the role of interferon pathways in the immune heterogeneity of asthma.

## Supplementary Information

**Additional file 1. **Additional tables.

**Additional file 2. **Additional figures.

## Data Availability

The datasets generated and/or analysed during the current study are available in the Sequence Read Archive (SRA) repository (GSE172495).

## Abbreviations

PBMCs: Peripheral blood mononuclear cells
SA: Severe asthma
scRNA-Seq: Single-cell RNA sequencing
CyTOF: Mass cytometry
HC: Healthy controls
NK: Natural killer
DC: Dendritic cells
IFN: Interferon
poly I: C: polyinosine–polycytidylic acid
dsRNA: Double-stranded RNA
YCAAD: Yale Center for Asthma and Airway Disease
SSC: Sodium citrate buffer
PFO: 1H,2H,2H-Perfluoro-1-octanol
FDR: False discovery rate
PCs: Principal components
PCA: Principal components analysis
UMAP: Uniform Manifold Approximation and Projection
GO: Gene ontology
tSNE: T-distributed stochastic neighbor embedding
STAMPs: Single-cell transcriptome attached to microparticles
JAK1: Janus kinase 1
lncRNA: Long non-coding RNA
*CMKLR1*: Chemerin chemokine-like receptor 1
ISGs: Interferon-stimulated genes
*PHF11*: Plant homeodomain finger protein 11
TSLP: Thymic stromal lymphopoietin
*MAPKs*: Mitogen-activated protein kinases
