# Supplementary figures and images for: Single-cell characterization of a model of poly I:C-stimulated peripheral blood mononuclear cells in severe asthma
Source: Respir Res. 2021 Apr 26;22:122. doi: 10.1186/s12931-021-01709-9 (PMC8074196; doi:10.1186/s12931-021-01709-9)

# Supplementary figure 1. IFNG expression in PBMCs

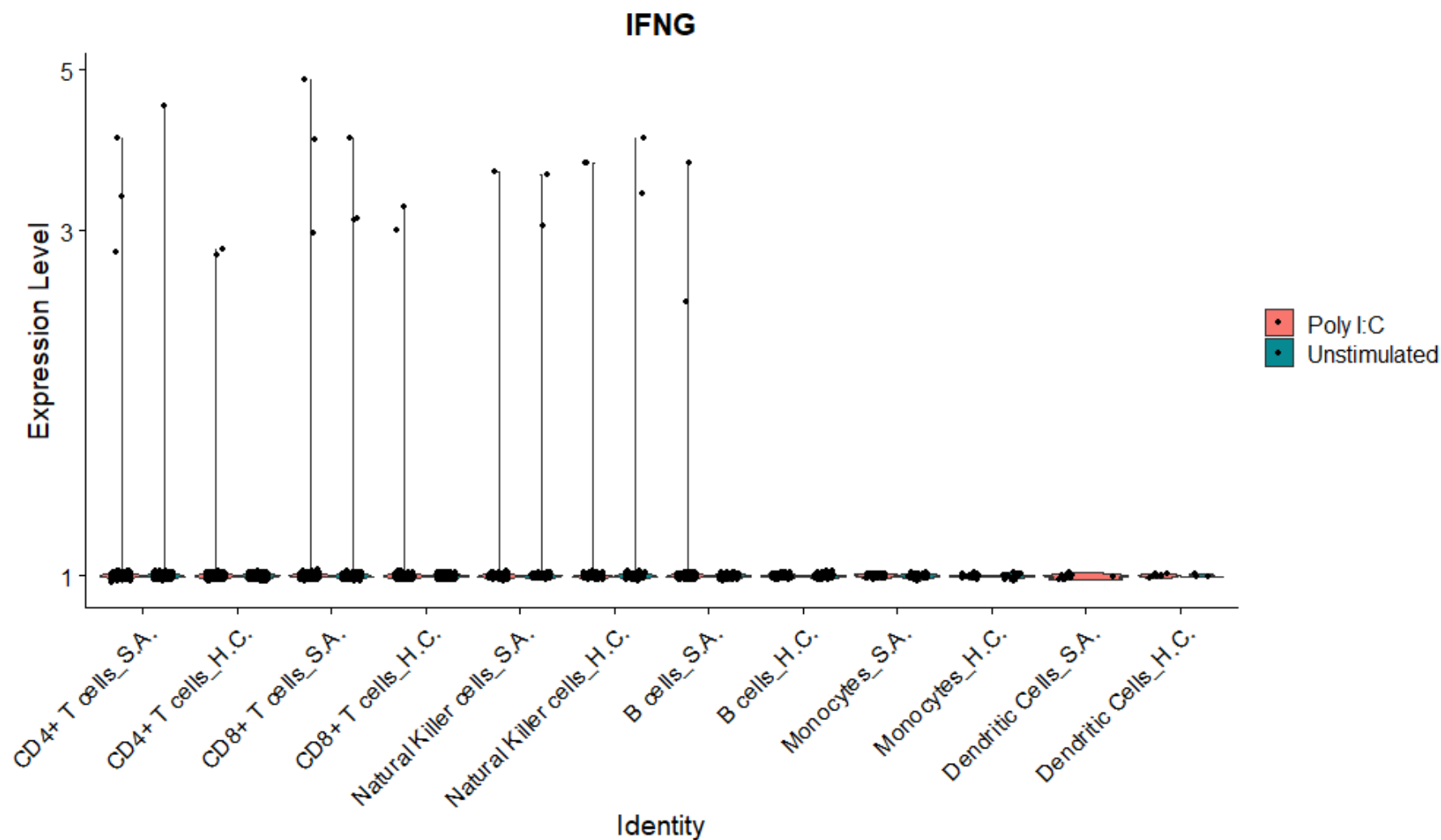

# Supplementary figure 2. All CyTOF clusters

**A.**

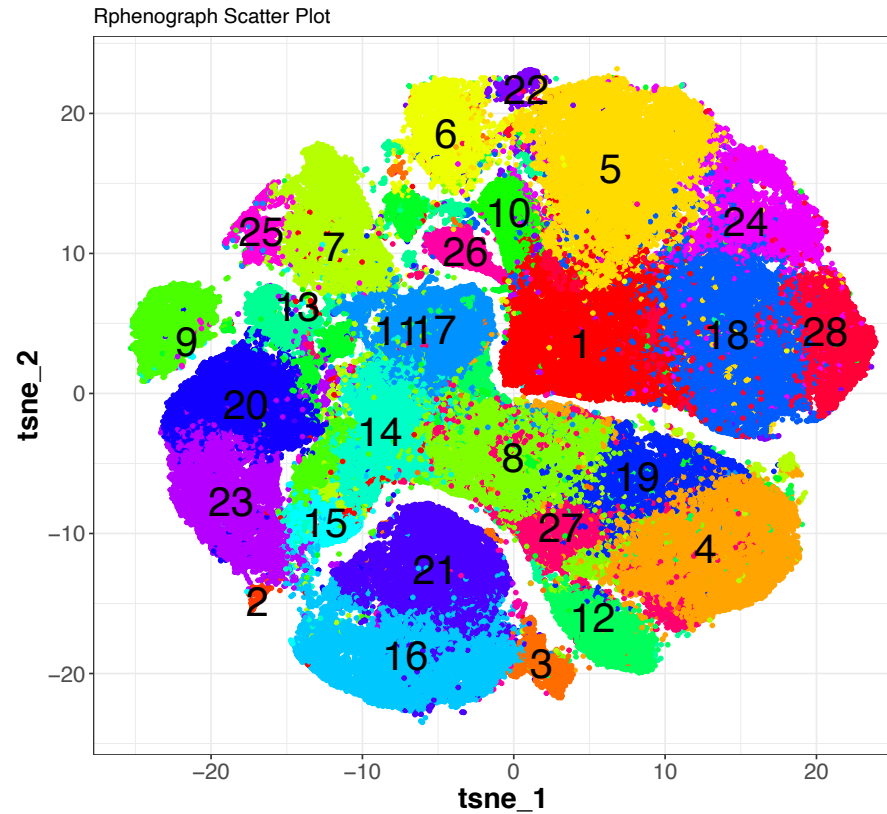

**B.**

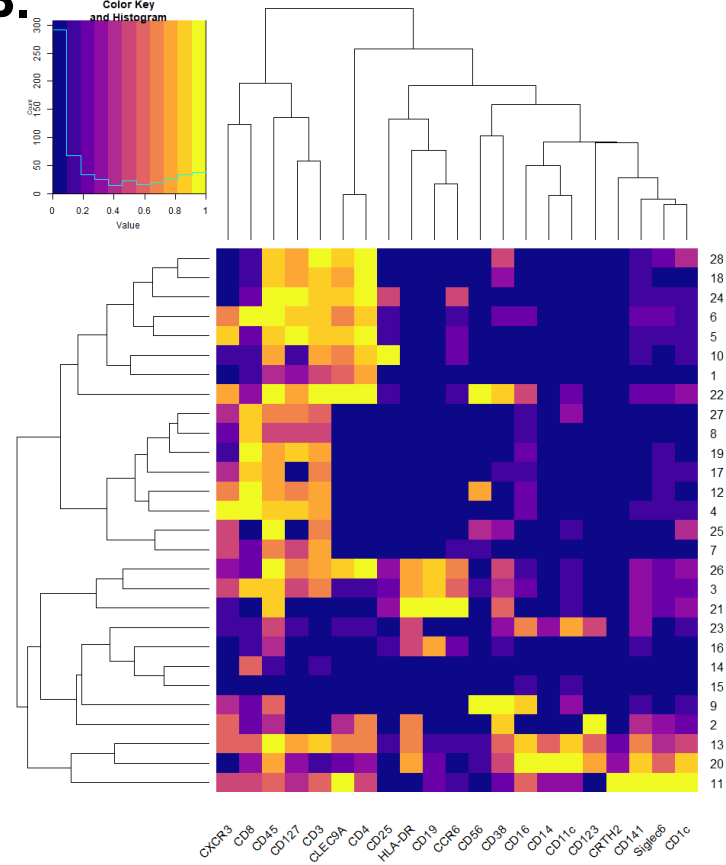

Supplement: Supplementary file 2 — Additional file 2. Additional figures. [file 12931_2021_1709_MOESM2_ESM.pdf]
